# Supplementary material for: The interactome of intact mitochondria by cross-linking mass spectrometry provides evidence for coexisting respiratory supercomplexes
Source: Mol Cell Proteomics. 2017 Dec 8;17(2):216–32. doi: 10.1074/mcp.RA117.000470 (PMC5795388; doi:10.1074/mcp.RA117.000470)
Supplement: Supplemental Data [file supp_RA117.000470_133922_0_supp_23978_fzffwf.zip › spectra_annotation/mito_DR_spectra_annotation/185-1-34-1-2-1.pdf]

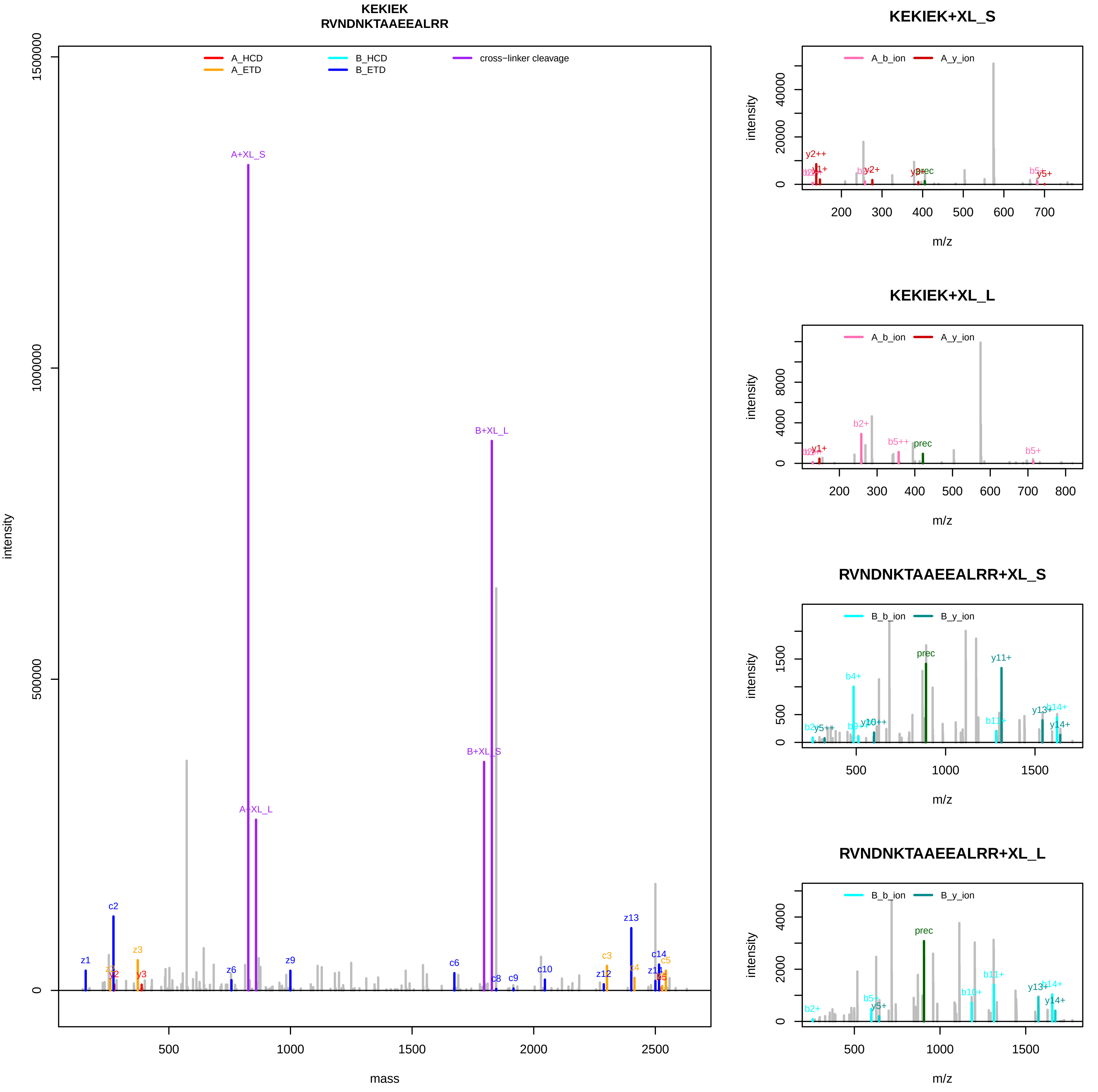
KEKIEK
RVNDNKTAAEEALRR
A_HCD
A_ETD
B_HCD
B_ETD
cross−linker cleavage
A+XL_S
A+XL_L
B+XL_L
B+XL_S
z1
c2
z2
y2
z3
y3
z6
z9
c6
c8
c9
c10
z12
z13
c3
c4
c5
c14
z14
z5
0
500000
1000000
1500000
intensity
500
1000
1500
2000
2500
mass
KEKIEK+XL_S
A_b_ion
A_y_ion
y2++
y1+
y2+
y3+
prec
b5+
y5+
0
20000
40000
200
300
400
500
600
700
m/z
intensity
KEKIEK+XL_L
A_b_ion
A_y_ion
b2+
y1+
b5++
prec
b5+
0
4000
8000
200
300
400
500
600
700
800
m/z
intensity
RVNDNKTAAEEALRR+XL_S
B_b_ion
B_y_ion
prec
b4+
y11+
y5++
y10++
b11+
y13+
b14+
y14+
0
500
1500
500
1000
1500
m/z
intensity
RVNDNKTAAEEALRR+XL_L
B_b_ion
B_y_ion
prec
b2+
b5+
y5+
b10+
b11+
y13+
b14+
y14+
0
2000
4000
500
1000
1500
m/z
intensity
